# 3D Tracking via Shoe Sensing

**DOI:** 10.3390/s16111809

**Published:** 2016-10-28

**Authors:** Fangmin Li, Guo Liu, Jian Liu, Xiaochuang Chen, Xiaolin Ma

**Affiliations:** 1 Department of Mathematics and Computer Science, Changsha University, Changsha 410022, China; 2 Key Laboratory of Fiber Optical Sensing Technology and Information Processing, Ministry of Education, School of Information Engineering, Wuhan University of Technology, Wuhan 430070, China; zeng_zhen@whut.edu.cn (G.L.); chenxc_whut@163.com (X.C.); maxiaolin0615@whut.edu.cn (X.M.); 3 Department of Electrical and Computer Engineering, Stevens Institute of Technology, Hoboken, NJ 07030, USA; jliu28@stevens.edu

**Keywords:** indoor localization, 3D positioning, inertial sensor, walking state classification

## Abstract

Most location-based services are based on a global positioning system (GPS), which only works well in outdoor environments. Compared to outdoor environments, indoor localization has created more buzz in recent years as people spent most of their time indoors working at offices and shopping at malls, etc. Existing solutions mainly rely on inertial sensors (i.e., accelerometer and gyroscope) embedded in mobile devices, which are usually not accurate enough to be useful due to the mobile devices’ random movements while people are walking. In this paper, we propose the use of shoe sensing (i.e., sensors attached to shoes) to achieve 3D indoor positioning. Specifically, a short-time energy-based approach is used to extract the gait pattern. Moreover, in order to improve the accuracy of vertical distance estimation while the person is climbing upstairs, a state classification is designed to distinguish the walking status including plane motion (i.e., normal walking and jogging horizontally), walking upstairs, and walking downstairs. Furthermore, we also provide a mechanism to reduce the vertical distance accumulation error. Experimental results show that we can achieve nearly 100% accuracy when extracting gait patterns from walking/jogging with a low-cost shoe sensor, and can also achieve 3D indoor real-time positioning with high accuracy.

## 1. Introduction

Nowadays, location-based service (LBS) has drawn considerable attention as it could provide a number of uses in various domains, such as entertainment, work and personal life, etc. Most LBS services, such as those in the conventional Garmin map and Google map, rely on GPS (Global Positioning System) to obtain accurate location information [1]. Although GPS location service has become very mature and can achieve high positioning accuracy, the performance of a GPS location service becomes poor for indoor environments due to a wide variety of physical signal blocks and potential sources of interference [2]. Thus, it is highly desirable to find an alternative that can provide stable positioning for indoor environments.

Recently, infrared-based and WiFi-based indoor positioning solutions have been explored. In general, these existing solutions are mainly focused on the positioning for 2D indoor LBS services. However, the complexity of the building floor structure and the restriction of 2D indoor positioning cannot always meet the demands of indoor LBS services. For example, firefighters in a burning building cannot effectively distinguish their own indoor positions or which floor they are on, making it very hard for them to perform effective rescues. It is crucial for them to have a 3D indoor positioning service to obtain their real-time locations. Another example is tracking hospital patient services. A hospital patient may be in critical condition at anytime and anywhere, and nurses and doctors need to be able to know all patients’ fine-grained indoor locations to provide immediate and effective treatments. Meanwhile, human activities are mainly concentrated in indoor environments, such as offices and malls, making the field of 3D indoor positioning/navigation a huge business opportunity. Therefore, how to achieve 3D indoor positioning with low-cost and non-invasive requirements has become a top topic in recent years.

Indoor positioning should consider not only the positioning accuracy but also the feasibility, resource consumption, and cost [3,4]. Although there are a number of solutions in this area, the indoor positioning problem has not been addressed satisfactorily due to a series of practical issues (e.g., device cost and deployment limits). Under such circumstances, it is important to develop a low-cost, low power consumption solution to achieve accurate 3D indoor positioning and tracking. Different from existing solutions, we propose the use of shoe sensors, which are low-cost inertial sensors attached to one of the user’s shoes to accurately localize the user in a 3D indoor environment. In this paper, we mainly focus on addressing the following three problems: (1) accurately extracting features from the shoe sensors according to the characteristics of human walking; (2) establishing human walking state classification model, which can distinguish the user’s walking status including normal walking, going upstairs, and going downstairs; and (3) relying on the walking model, reducing the accumulation of positioning errors while walking.

Specifically, the following contributions are made in this work:

- We propose a solution using 3D shoe sensors, inertial sensor attached to the user’s shoes that can accurately localize the user in 3D indoor environments.
- A short-time energy-based mechanism has been proposed to extract gait information while the user is walking.
- We design a walking state classification model that can distinguish the user’s walking status including normal walking, going upstairs, and going downstairs. The classified walking status can be further used to reduce 3D positioning errors.
- Extensive experiments demonstrate that the proposed low-cost shoe sensing-based 3D indoor positioning solution can perform real-time localization with high accuracy.

The remainder of this paper is organized as follows. We describe the related work in Section 2. In Section 3, we present the methodology of the proposed shoe sensing-based 3D tracking. We evaluate the performance of our system in Section 4. Finally, conclusions are given in Section 5.

## 2. Related Work

Recently, with the development of MEMS inertial sensor devices, inertial sensor-based navigation solutions are becoming more and more popular in indoor localization scenarios [5]. Specifically, inertial sensor-based navigations can be categorized as either stepping-based or strap-down-based navigation systems [6].

Stepping-based navigation systems use step information (e.g., the number of walking steps and step length) to detect pedestrians’ positions [6,7,8]. For example, in [6] the users carry an inertial sensor in their pockets, and the system can calculate the inertial sensor’s pitch angle and further estimate the step length. However, this method assumes that different people have the same step length, making it hard to achieve accurate localization in practice.

Strap-down-based navigation systems integrate acceleration readings twice to get the walking distance. With the aid of a compass and gyroscope, the system can also capture the walking direction. Existing solutions can be divided into two categories: one is carrying the sensor at the user’s waist, and the other is attaching sensors to the shoes.

Inertial sensors fixed at the waist could be used to detect the user pelvis’s vertical displacement and estimate the length of each step [9,10]. However, there are different walking characteristics due to people’s various height, weight, age, etc. In order to improve the accuracy of the estimated step length, step length for walking, and step frequency, Shin et al. [11] use personal training historical data to study the personal characteristics of each user. Due to the sensor’s internal bias, there is an accumulation drift problem over time [1]. To address this problem, Shih et al. [1] use a mathematical model of simple harmonic motion to calculate each step’s length. However, this method still does not work well due to the different height of each individual.

There are also several studies using shoe sensors to estimate a pedestrian’s position. According to the characteristics of human walking (e.g., the sensor velocity should be zero when the foot is on the ground), zero speed correction (zero-velocity update, ZUPT) [12,13,14,15,16,17,18,19] is proposed to correct the accumulation drift. However, these solutions mainly focus on 2D positioning. In addition, Li et al. [15,19,20] use a Kalman filter or complementary filter to calibrate the inertial sensor data, which has high algorithm complexity. Moreover, Placer et al. [17] use a camera sensor attached to the shoes to eliminate measurement error and improve the localization accuracy. Although a camera-based solution can effectively improve the accuracy of step estimation, the camera-based sensor leads to complicated data acquisition and consumes more power.Madgwick et al. [21,22] use gradient descent algorithm to eliminate gyro errors. Additionally, Nilsson et al. [14] uses an expensive inertial sensor unit (i.e., over $700 per unit).Nilsson et al. [23,24,25,26] also use an expensive sensor placed under the heel, and such solutions mainly focus on 2D indoor localization. Ojeda et al. [27] proposed ZUPT-based 3D positioning, but this does not apply any amendment to fix sensor measurement errors.

Additionally, some studies (e.g., [27,28,29,30]) use the inertial sensors of smartphones to complete interior 2D positioning. However, these approaches rely on the aid of other methods such as WiFi to achieve better positioning performance. These approaches increase the complexity of the positioning system and WiFi signals are not always available in indoor environments.

## 3. Methodology

### 3.1. Gait Information

The estimated distance error will keep accumulating due to the drift of inertial sensor readings [31]. According to the characteristics of human walking, we can eliminate the accumulated error of distance by using zero reference points from gait information. The zero reference points are the moments when the user’s feet step on the floor while walking. The gait information can be derived by using the short-time energy, which is usually applied on the audio signal, on acceleration. Also, we try to find a feasible position for fixing the sensors by comparing the energy of the signal.

#### 3.1.1. Fixed Position Selection

The walking patterns and the gait information of every step should be clear from the sensor reading [32]. We observe that the walking pattern and the gait information are more stable with the sensors fixed on the foot compared to other places.

**Figure 1:**
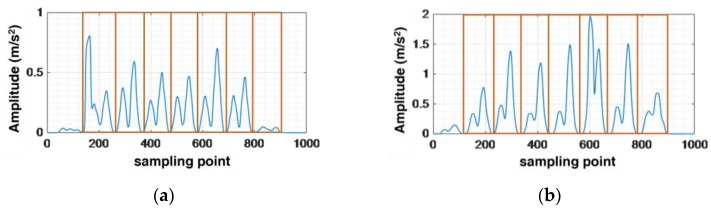

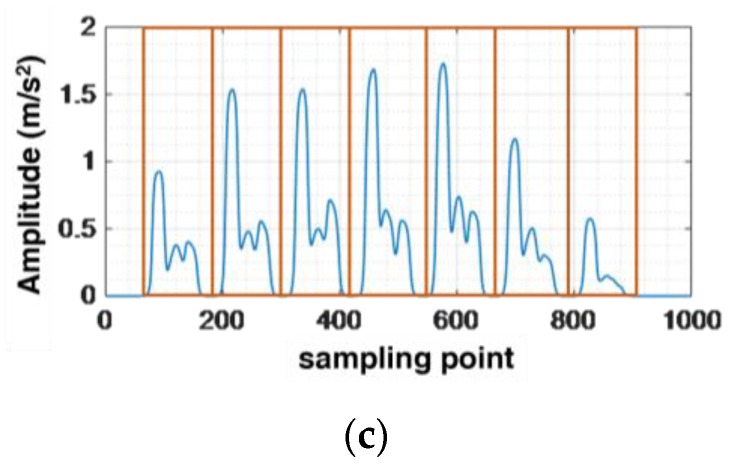
Amplitude of raw acceleration with sensor fixed on different position. (**a**) Fixed on thigh; (**b**) fixed on legs; (**c**) fixed on foot.

**Figure 2:**
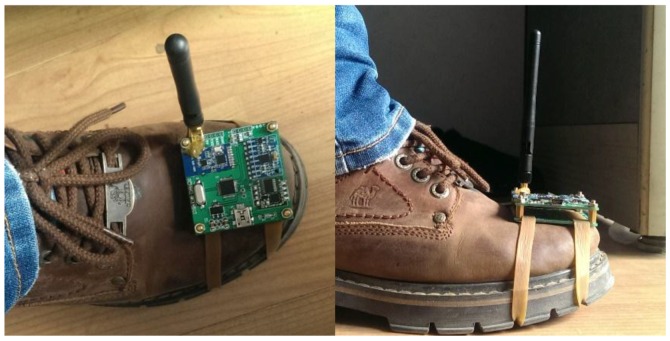
Inertial sensor fixed on foot.

Figure 1 shows an example of the energy of acceleration (i.e., $x=\sqrt{accX^{2}+accY^{2}+accZ^{2}}$ − *g*) on all three axes while a person is walking seven steps with the inertial sensors fixed in different positions (i.e., thigh, leg, and foot). We can see that the energy varies in the duration of every step. What is more, the energy of the acceleration will decrease to zero when the foot steps on the floor. Comparing Figure 1a,b with Figure 1c, we can see that the walking pattern of every step is much more stable with the sensor fixed on the foot. Meanwhile, the duration of zero velocity points is longer than the other two fixed positions. Therefore, we choose the foot as the fixed position of the sensors. While conducting experiments, we fixed the sensors as shown in Figure 2.

#### 3.1.2. Gait Information Extraction

The gait information can be derived by comparing the energy fluctuation on all three axes of the accelerometer. We observe that the energy of acceleration varies along with the patterns of the foot. That is to say, while the foot is in the air the energy of acceleration is high; while the foot is stepping on the floor the energy of acceleration is decreased to zero. Therefore, we can extract the gait information by using a threshold of the short-time energy of the acceleration.

**Figure 3:**
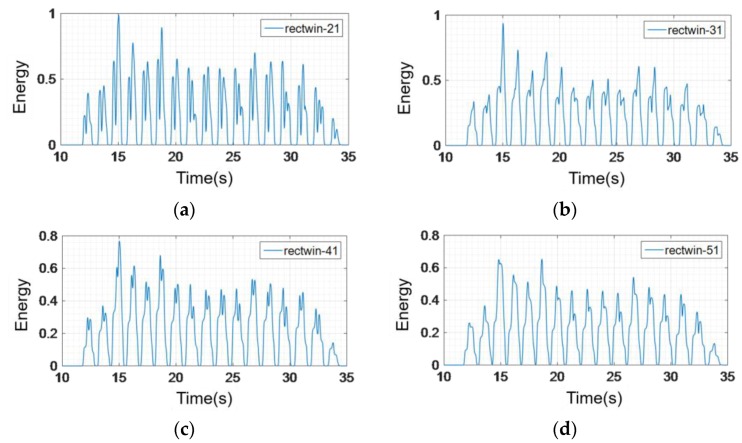
Energy waveforms with different window sizes: (**a**) window size of 21; (**b**) window size of 31; (**c**) window size of 41; (**d**) window size of 51.

The acceleration of human walking varies on three axes. In order to take the energy fluctuation on all three axes into consideration, we use the amplitude of acceleration to extract the gait information. The amplitude can be calculated as follows:

$$x=\sqrt{accX^{2}+accY^{2}+accZ^{2}}-g,$$

where accX,accY,accZ are the acceleration on the *X*, *Y*, and *Z* axis, respectively, and *g* is gravity. Therefore, the short-time energy signal of human walking can be derived as:

$$E_{n}={\sum_{m=n-N+1}^{n}\left[x(m)w\left(n-m\right)\right]}^{2},$$

where *n* is the position of the center of the sliding window and *N* is the length of the window. *N* is critical for controlling the fluctuation of the energy. Figure 3 shows an example of the short-time energy with different sliding window lengths. We can see that the short-time energy wave is smoother with a longer window length.

According to the foregoing conclusions, the short-time energy of human walking will decrease to zero while the foot steps on the floor. Thus we can extract the gait information by setting a threshold $E_{T}=0.05$ of energy. The reference points can be determined as:

$$stationary\left(t\right)=\{\begin{array}{c} 1\;E\left(t\right)<E_{T}\\ 0\;E\left(t\right)\geq E_{T} \end{array}.$$

**Figure 4:**
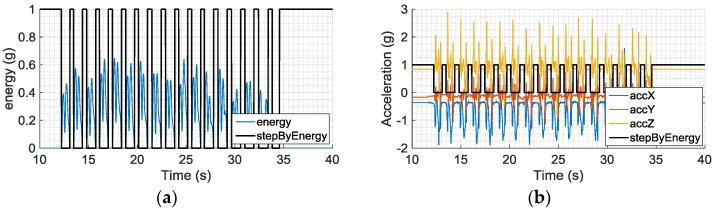
Waveform of gait signal: (**a**) waveform of energy and gait; (**b**) acceleration and gait.

Figure 4 shows an example of gait information (i.e., reference points) extraction using our gait extraction method.

### 3.2. Posture Correction Based on Gait Information

Quaternion is a hyper-complex number, which can be expressed as:

$$Q\left(q_{0},q_{1},q_{2},q_{3}\right)=q_{0}+q_{1}i+q_{2}j+q_{3}k.$$

**Figure 5:**
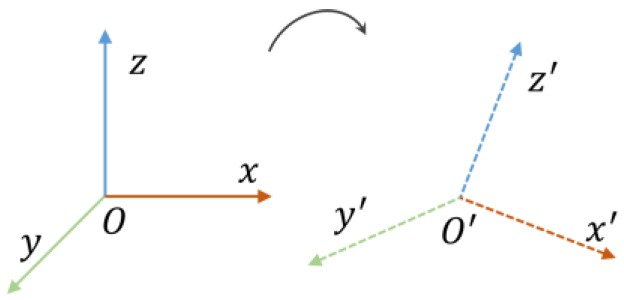
Reference coordinate system and sensor coordinate system.

Figure 5 shows an example of coordinate system alignment with a quaternion. *O* is the reference coordinate system and *O*’ is the coordinate system of the inertial sensor. In this paper, the system needs to derive the quaternion that describes the attitude of the inertial sensor relative to the reference coordinate system.

While the foot is stepping on the floor, the acceleration data is stable. Thus we can estimate the initial posture of the sensors by using a method proposed in previous research [22].

**Figure 6:**
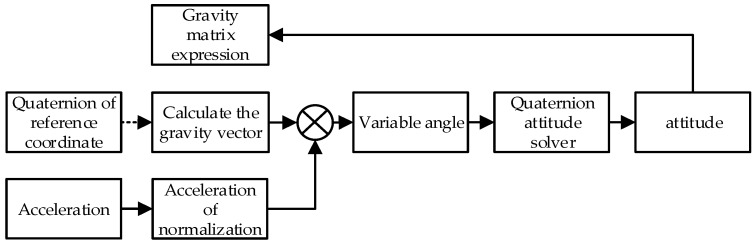
Posture initialization process.

Figure 6 shows the process of our posture initialization method. Supposing the initial quaternion of the reference coordinate system is $q_{0}=[1,0,0,0]$, we can calculate the gravitational acceleration vector $a_{R}={\left[0,0,1\right]}^{T}$ of the reference coordinate system.

Following this direction, since the quaternion rotation matrix can be derived as:

$$C_{b}^{R}=\left[\begin{array}{ccc} q_{0}^{2}+q_{1}^{2}-q_{2}^{2}-q_{3}^{2} & 2\left(q_{1}q_{2}-q_{3}q_{0}\right) & 2\left(q_{1}q_{3}+q_{0}q_{2}\right)\\ 2\left(q_{1}q_{2}+q_{0}q_{3}\right) & q_{0}^{2}-q_{1}^{2}+q_{2}^{2}-q_{3}^{2} & 2\left(q_{2}q_{3}-q_{0}q_{1}\right)\\ 2\left(q_{1}q_{3}-q_{0}q_{2}\right) & 2\left(q_{2}q_{3}+q_{0}q_{1}\right) & q_{0}^{2}-q_{1}^{2}-q_{2}^{2}+q_{3}^{2} \end{array}\right],$$

the gravity in the sensor coordinate system can be calculated by rotating the gravity in the reference coordinate system:

$$g_{b}=C_{b}^{R}\times a_{R}$$

$$g_{b}=\left(\begin{array}{c} g_{bx}\\ g_{by}\\ g_{bz} \end{array}\right)=\left(\begin{array}{c} 2\left(q_{1}q_{3}-q_{0}q_{2}\right)\\ 2\left(q_{2}q_{3}+q_{0}q_{1}\right)\\ q_{0}^{2}-q_{1}^{2}-q_{2}^{2}+q_{3}^{2} \end{array}\right).$$

The raw acceleration from sensor readings can be described as:

$$g_{R}={\left[\begin{array}{ccc} a_{x} & a_{y} & a_{z} \end{array}\right]}^{T},$$

which can be normalized as:

$${g\prime }_{R}=\frac{g_{R}}{\sqrt{a_{x}^{2}+a_{y}^{2}+a_{z}^{2}}}.$$

Therefore, we can derive the quaternion as:

$$Q_{n}\left(q_{n0}+q_{n1}+q_{n2}+q_{n3}\right)=v_{n}P,$$

where ***P*** is the solution process of the quaternion, $v_{n}=a_{R}\times {g\prime }_{R}$ is the vector product of, and (***R*** = 1, 2, 3, …, *n*). The calculated quaternion of *Q* expression approaches the acceleration of gravity, so we can calculate the $g_{b}$ of the gravitational acceleration vector. The posture can be initialized by repeating the above steps.

**Figure 7:**
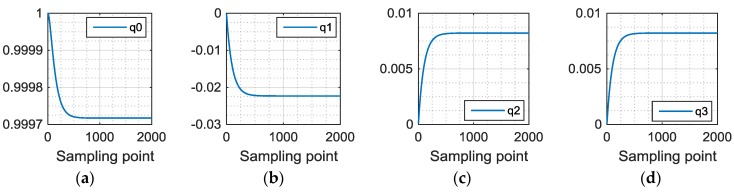
Convergent process. (**a**–**d**) describes the convergent process of the four elements in quaternion.

The above calculation process is convergent. The estimated coordinate system of the inertial sensor will converge to the real attitude. Figure 7 shows an example of the convergent process of deriving the quaternion while we conducting experiments. We find that our method will converge in about 4 s (i.e., 400 points).

Madgwick et al. [22] uses a gradient manner to eliminate the direction error. Following this method, the acceleration while the foot is stepping on the floor can be used as a reference value to estimate the error between the sensor coordinate system and the reference coordinate system, since it is more stable [22]. Thus, we can correct the drift error of the gyroscope by using the estimated error.

**Figure 8:**
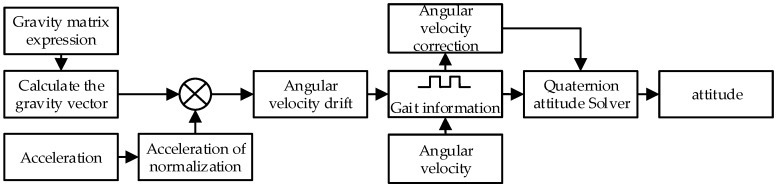
Posture estimate based on gait information.

Figure 8 shows the work flow of gait information based posture estimation. The gait information based posture estimation and the attitude initialization are similar. The vector product of $g_{b}$ and ${g\prime }_{R}$ is angular velocity error *e*. The larger *e* is, the greater the angular velocity error will be. Combined with gait information, the gyro angular velocity can be expressed as:

$$gyro^{\prime }=gyro-Kp\cdot e\cdot stationary,$$

where *gyro* is the angular velocity vector, *Kp* is the gain error coefficient of system. We use the fourth-order Runge–Kutta method to update the quaternion. The differential of the quaternion is defined as:

$$Q^{\prime }=\frac{1}{2}w\left(t\right)*Q\left(t\right).$$

At time $t_{0}$ it is:

$$Q\left(t_{0}\right)=Q_{0}.$$

The corresponding differential equation is:

$$\left[\begin{array}{c} {\dot{q}}_{0}\left(t\right)\\ {\dot{q}}_{1}\left(t\right)\\ {\dot{q}}_{2}\left(t\right)\\ {\dot{q}}_{3}\left(t\right) \end{array}\right]=\frac{1}{2}\left[\begin{array}{cccc} 0 & -w_{x} & -w_{y} & -w_{z}\\ w_{x} & 0 & w_{z} & -w_{y}\\ w_{y} & -w_{z} & 0 & w_{x}\\ w_{z} & w_{y} & -w_{x} & 0 \end{array}\right]\left[\begin{array}{c} q_{0}\left(t\right)\\ q_{1}\left(t\right)\\ q_{2}\left(t\right)\\ q_{3}\left(t\right) \end{array}\right].$$

The quaternion can be derived by using the fourth-order Runge–Kutta method as follows:

$$Q_{n+1}=Q_{n}+\frac{h}{6}\left(k_{1}+2k_{2}+3k_{3}+k_{4}\right)$$

$$k_{1}=f\left(w_{n},Q_{n}\right)$$

$$k_{2}=f\left(w_{n}+\frac{h}{2},Q_{n}+\frac{h}{2}k_{1}\right)$$

$$k_{3}=f\left(w_{n}+\frac{h}{2},Q_{n}+\frac{h}{2}k_{2}\right)$$

$$k_{4}=f\left(w_{n}+h,Q_{n}+hk_{3}\right),$$

where $w_{x},w_{y},w_{z}$ are the raw angular velocities of inertial sensor and *h* is the actual sampling interval. Updating the quaternion in real time will gradually result in losing quaternion specification properties. So we must normalize the quaternion as follows:

$$q_{i}=\frac{q_{i}}{\sqrt{{\hat{q}}_{0}^{2}+{\hat{q}}_{1}^{2}+{\hat{q}}_{2}^{2}+{\hat{q}}_{3}^{2}}}\;\left(i=1,2,3\right),$$

where ${\hat{q}}_{0},{\hat{q}}_{1},{\hat{q}}_{2},{\hat{q}}_{3}$ are the quaternion values of updating.

### 3.3. Eliminate Cumulative Error Based on Gait Information

**Figure 9:**
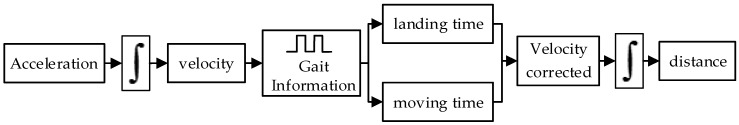
Accumulated error elimination.

The gait information can not only be used as the basis of gyroscope error elimination, but also can be the reference point for eliminating cumulative error. As shown in Figure 9, Following the idea of Yun and Han et al. [20,33], the accumulated error in acceleration can be eliminated based on the zero velocity reference point and the linear drift characteristics of inertial sensors.

In our experiments, we fix the sensor on the foot of the user with its *x* axis along the user’s toe direction, which is also the moving direction of the user. Thus, the moving speed of the user can be expressed as:

$$v\left(T\right)=v\left(0\right)+\sum_{i=0}^{Tk}\frac{1}{k}acc_{x}\left(i\right),$$

where ***k*** is the sample rate of the sensors. In our experiments, we found that the sampling rate can range from 40 to 100 Hz without affecting the system performance significantly. This range covers most of the maximum accelerometer sampling rate of current smartphones. Therefore, we focus on other factors that are apropos to the performance of the system and set the sample rate at 100 Hz during our experiments.

**Figure 10:**
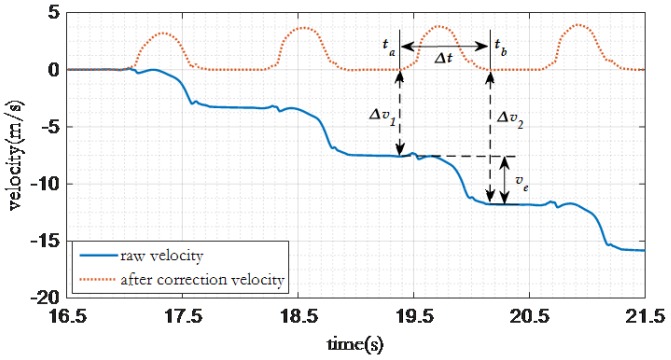
Based on cumulative error gait elimination.

As shown in Figure 10, the accumulated velocity error from $t_{a}$ to $t_{b}$ can be calculated as follows:

$$v_{e}=\Delta v_{2}-\Delta v_{1},$$

then the gradient of accumulated velocity error during this period is:

$$\tilde{e}=\frac{\Delta v_{2}-\Delta v_{1}}{t_{b}-t_{a}}.$$

According to previous work [33], if the gradient of accumulated velocity error during this period is constant, then velocity at time T can be derived as:

$$V\left(T\right)=V\left(ak-1\right)+\sum_{i=ak}^{bk}\frac{1}{k}acc_{x}\left(i\right)-\frac{\Delta v_{2}-\Delta v_{1}}{t_{b}-t_{a}}\times \frac{i-ak}{k}.$$

When the foot is landing, the velocity should be zero:

V(ak−1)=0,

then the velocity can be expressed as:

$$V\left(T\right)=\{\begin{array}{ll} \sum_{i=a_{n}k}^{b_{n}k}\frac{1}{k}acc_{x}\left(i\right)-\tilde{e}\frac{i-a_{n}k}{k} & stationary=1\\ 0 & stationary=0 \end{array},$$

where $\hat{e}$ is the accumulated error gradient of each step, $a_{n}$ is the start time of every step, and $b_{n}$ is the ending time of every step. The human walking distance can be calculated by integrating the corrected velocity *V(T)*. Assuming the initial distance is zero, the distance of a certain axis can be expressed as:

$$S\left(T\right)=\sum_{i=0}^{TK}\frac{1}{k}V\left(i\right).$$

### 3.4. Eliminate Vertical Distance Error Based on Gait Information

#### 3.4.1. Build and Design a Model of State

**Figure 11:**
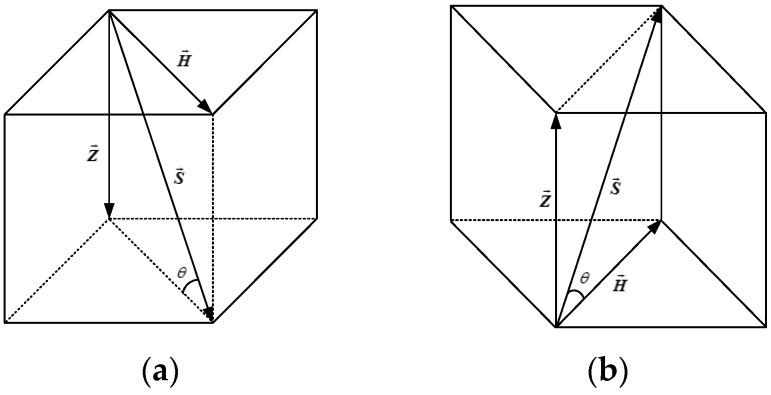
Walking state classification model: (**a**) downstairs; (**b**) upstairs.

In this section, we focus on how to distinguish between the body’s normal walking upstairs, downstairs, and along a plane (normal walking, jogging). We found an effective way to distinguish between the different walking patterns based on the characteristics of human walking. Figure 11 shows an example of the walking model while the user is walking upstairs and downstairs, respectively.

Each step can be abstracted as a normal walking vector $\vec{S}$, which is the sum of $\vec{H}$ and $\vec{Z}$. While walking in the horizontal plane, θ can be expressed as:

$$\theta =\arccos\frac{\vec{Z}}{\left|\vec{S}\right|},$$

where θ stands for the degree of change on the *z* axis while the user takes a random step. However, θ is different between different users, since the walking pattern varies greatly among different users. In order to distinguish between walking along a plane, upstairs, and downstairs, we design a novel mathematical model:

$$\theta^{\prime }=\frac{\theta }{\left|\vec{H}\right|},$$

where θ is the angle change of a unit horizontal distance. The main purpose of this method is to eliminate the error caused by individual walking characteristics by normalizing the distance changes on the ***z*** axis.

**Figure 12:**
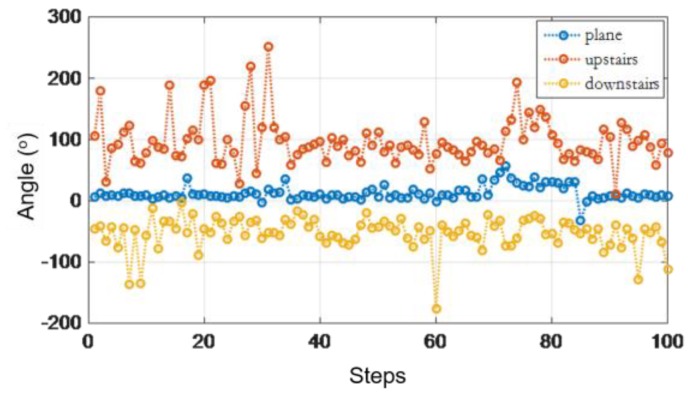
$\theta^{\prime }$ angle contrast.

Figure 12 shows an example of $\theta^{\prime }$ when a user walks in different environments. We can observe that the $\theta^{\prime }$ is different when the user walks along a plane, upstairs, or downstairs. It is easy to distinguish the state of human walking by using a threshold of the average $\theta^{\prime }$, which is shown in Table 1.

#### 3.4.2. Eliminate Vertical Distance Error

**Figure 13:**
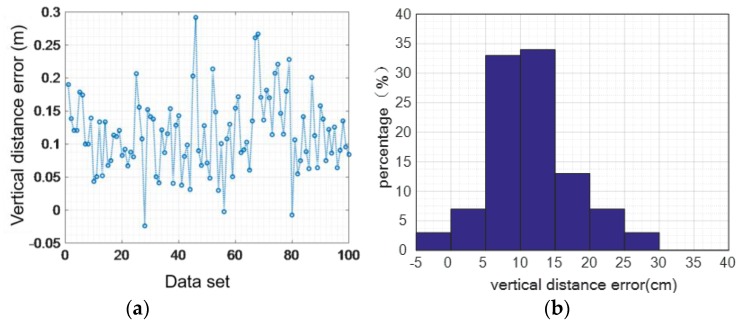
The vertical distance error while walking: (**a**) 100 sets of walking data; (**b**) vertical distance error.

**Figure 14:**
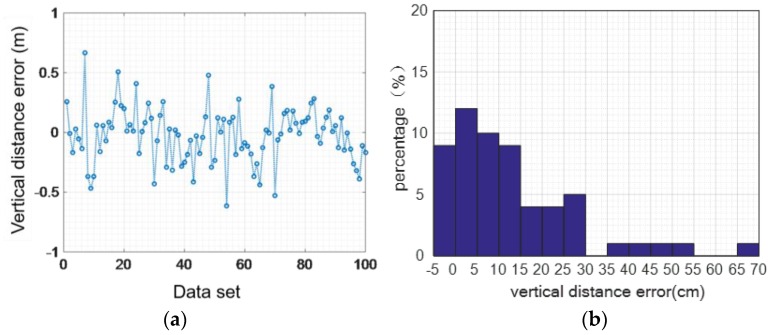
The vertical distance error while running: (**a**) 100 sets of running data; (**b**) vertical distance error.

**Figure 15:**
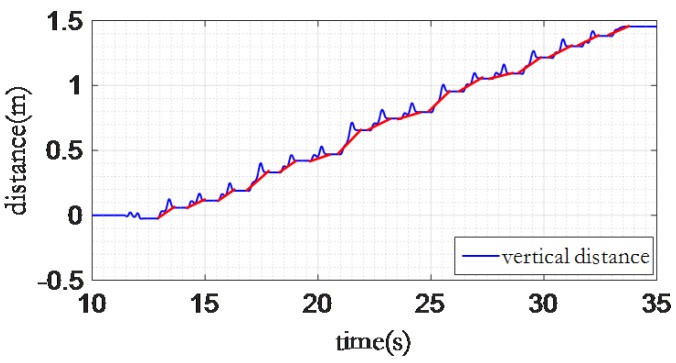
Vertical error accumulation.

Although we can eliminate the accumulated error from the speed level, the error in the vertical direction cannot be completely eliminated, which will lead to height calculation errors. Figure 13, Figure 14 and Figure 15 show the height error from 100 sets of walking and running data, respectively. While the user is walking, the maximum vertical distance error is 0.291 m, the average absolute error is 0.1186 m, and the variance of the error is 0.0582 m.

While the user is running, the maximum vertical distance error is 0.66 m, the average absolute error is 0.17 m, and the mean square deviation 0.22 m.

The error of indoor 3D localization is partly introduced by the accumulated error on the *Z* axis. Figure 15 shows an example of the displacement on the *Z* axis while the user walks on a flat floor. We can observe that the displacement on the *Z* axis accumulates after every step.

In order to eliminate the foregoing accumulated error, we propose a strategy based on the average human walking pattern and the gait information.

**Figure 16:**
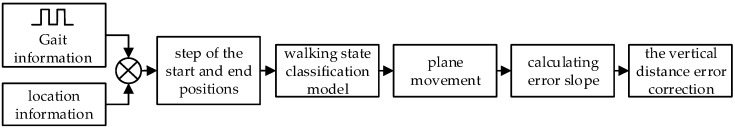
Plane vertical distance error elimination process.

**Figure 17:**
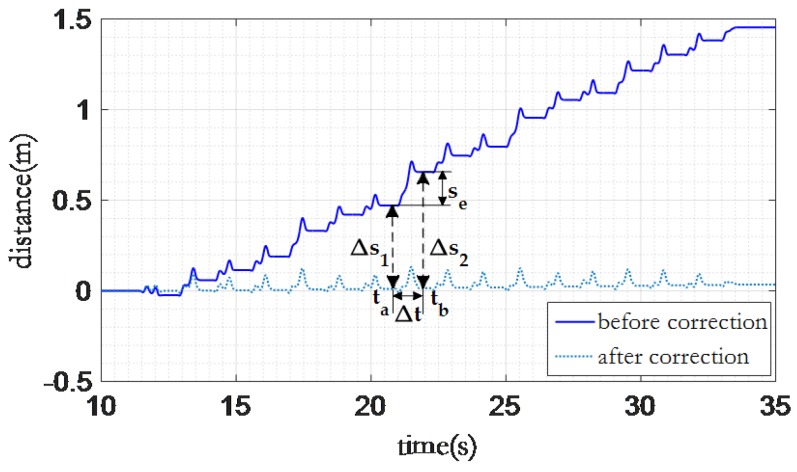
The vertical distance error elimination.

Figure 16 shows the workflow of our error elimination strategy. The start and end point of each step can be derived from the gait information. In addition, we can determine whether the user is walking on a flat floor by using the walking pattern model. Then we can eliminate the accumulated error on the *Z* axis through our strategy as shown in Figure 17.

The accumulated error from $t_{a}$ to $t_{b}$ is:

$$s_{e}=\Delta s_{2}-\Delta s_{1}.$$

Then we can calculate the distance error gradient as:

$${\tilde{e}}_{s}=\frac{\Delta s_{2}-\Delta s_{1}}{t_{b}-t_{a}}.$$

In this step, we assume the distance error gradient is constant, thus the moving distance on the *Z* axis while taking a step can be derived as:

$$S_{z}\left(T\right)=S_{z}\left(ak-1\right)+\sum_{i=ak}^{bk}\left(\frac{1}{k}V_{z}\left(i\right)-{\tilde{e}}_{s}\frac{i-ak}{k}\right),$$

and the moving distance on *Z* axis can be estimated by using:

$$S_{z}\left(T\right)=\{\begin{array}{ll} S_{z}\left(a_{n}k-1\right)+\sum_{i=a_{n}k}^{b_{n}k}\left(\frac{1}{k}V_{z}\left(i\right)-{\tilde{e}}_{s}\frac{i-a_{n}k}{k}\right) & stationary=1\\ S_{z}\left(a_{n}k-1\right) & stationary=0 \end{array}.$$

## 4. Evaluation

### 4.1. Building a System Platform and Experimental Settings

#### 4.1.1. Building a System Platform

**Figure 18:**
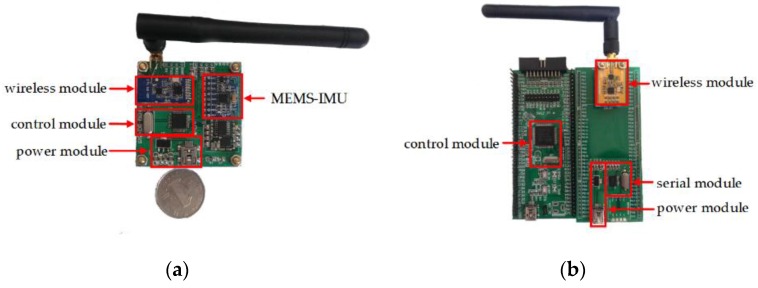
Collection node and network node: (**a**) collection node; (**b**) network node.

The acquisition and network nodes are the main hardware modules in our system. The collection nodes focus on packing and sending data from a gyroscope and an accelerometer to the network node. Then the data will be delivered to the PC monitoring client via the serial port after parsing by the network node for further processing and displaying. Figure 18 shows the collection node and network node of our system. The size of the nodes is designed as 5 cm × 5 cm for convenience.

#### 4.1.2. Experimental Environment Settings

**Figure 19:**
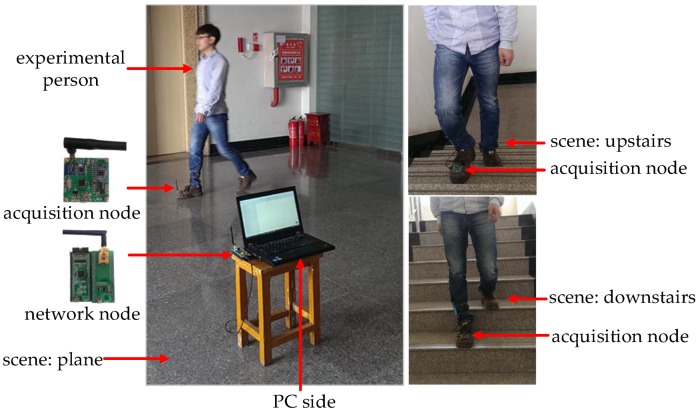
Experimental scene.

Since the MEMS inertial sensor is hardly affected by the external environment, we do not need to consider the effect of other external factors. Considering the difference between 2D and 3D localization is height information, the main experimental scene can be divided into two classes: moving in the horizontal plane (normal walking and jogging) and climbing stairs. Figure 19 shows our experimental scene.

### 4.2. Experimental Results and Analysis

#### 4.2.1. Gait Information Extraction Experiments

The accuracy of the gait information extraction is critical for the accuracy of 3D indoor localization. We focus on extracting gait information of four statuses, normal walking, running, going upstairs, and going downstairs. Then we will verify the accuracy of the gait information extraction based on the short-time energy of these statuses.

**Figure 20:**
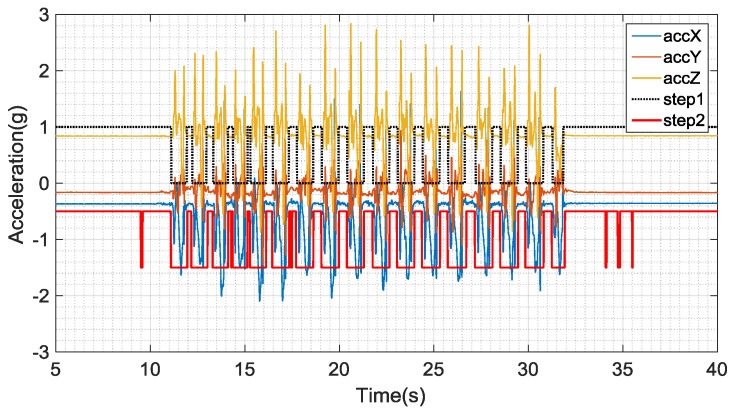
Comparative information extraction gaits.

In order to accurately extract the gait information and reduce the decision delay, the threshold should be set as small as possible while using both methods. Figure 20 shows an example of extracting gait information. We can observe that the extracted gait information while walking on a flat floor is fine with our short-time energy method, yet abnormal with the acceleration magnitude-based method. The problem arises when extracting the gait information of the other three statuses.

For the four different states, in this paper we perform experiments under different scenarios. The experimental field of normal walking and jogging is conducted in the corridor of a school building. For the benchmark experiments, normal walking and jogging activities are conducted along a straight line with a distance of 19.2 m. Normal walking and jogging are performed in 20 rounds each (i.e., back and forth). The tests of walking upstairs and downstairs were conducted on the stairs of the school building. To facilitate testing and statistical analysis of the experimental data, walking upstairs and downstairs were also conducted in 20 rounds.

**Figure 21:**
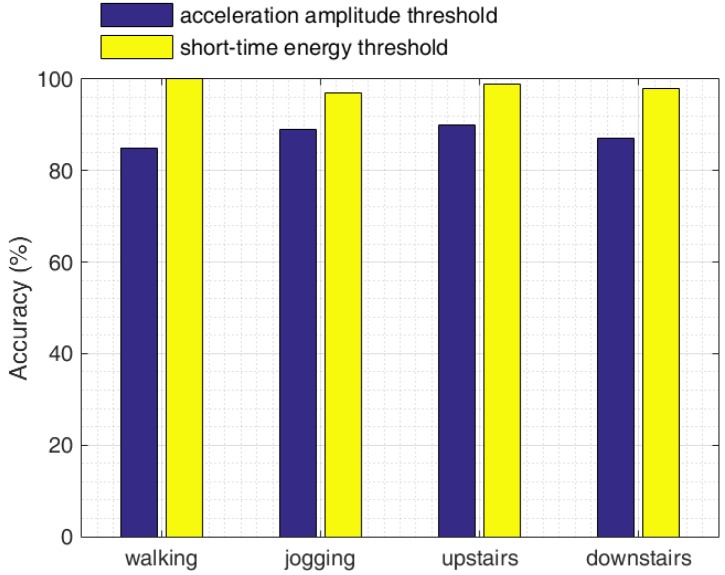
Gait information extraction accuracy.

Figure 21 shows the accuracy of gait information extraction for all four statuses with our short-time energy method and the acceleration amplitude-based method. We can see that our method provides sufficient accuracy to extract those four common moving activities.

#### 4.2.2. Walking State Classification Model Experiment

According to the judgement model of walking state presented in this paper, it can distinguish three kinds of states, a horizontal plane of movement (normal walking, jogging), going upstairs, and going downstairs. We mainly verify the accuracy of the judgment mentioned before with the collected data containing normal walking, jogging, going upstairs, and going downstairs as four states. We regard both jogging and normal walking as the same moving state. Specifically, we extract the experimental data of 100 steps of each state to analyze the statistics of walking state classification.

**Figure 22:**
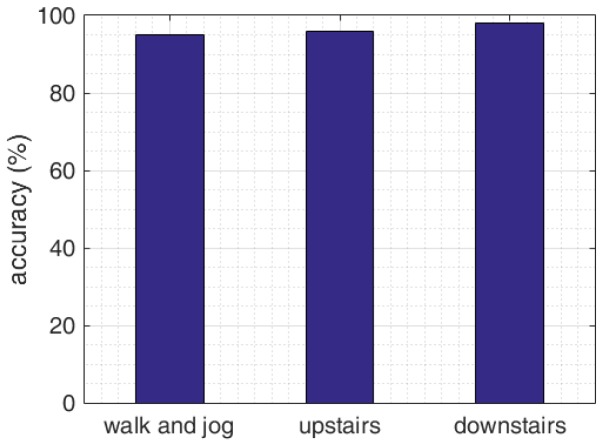
Traveling state determining statistical accuracy.

It can be seen from the Figure 22 that using the mathematical model designed in this paper, the judgement for three states can achieve above 95% accuracy and the mathematical model can judge the walking state effectively. Thus, it provides a reliable precondition for eliminating the error of vertical distance.

#### 4.2.3. Error Elimination in the Vertical Direction

**Figure 23:**
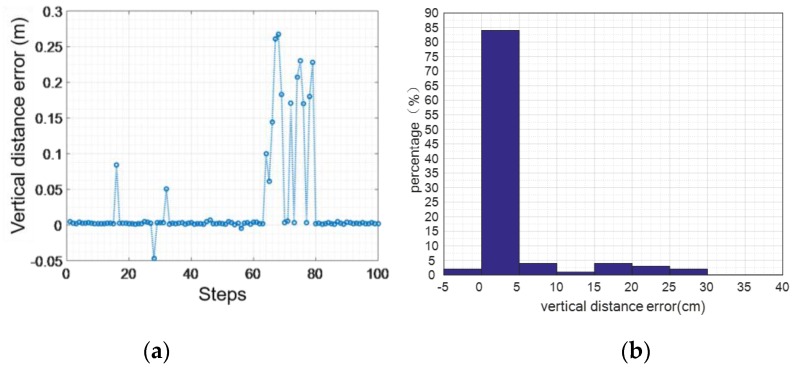
Walking normally: vertical distance error elimination: (**a**) distance error; (**b**) error percentage.

The method designed in this paper eliminates errors of the vertical direction when moving in the perpendicular plane based on the judgement model of walking state. We assessed the two states of normal walking and jogging by getting statistical data for 100 steps. As shown in Figure 23, for the walking activity, the maximum distance error is 0.26 m, the mean distance of the absolute value error is 0.02 m, and the distance mean square error of the absolute value is around 0.06 m.

**Figure 24:**
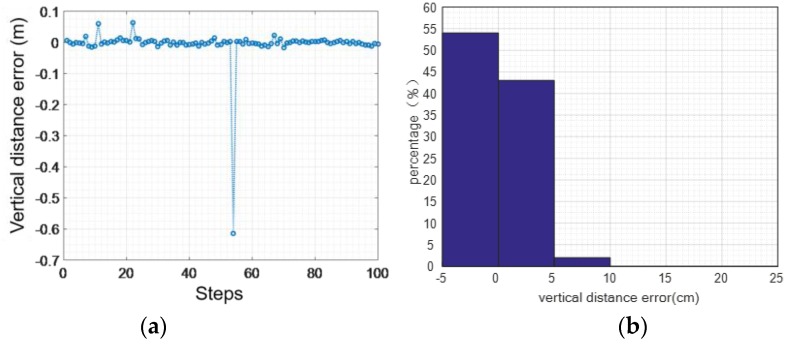
Jogging: vertical distance error elimination: (**a**) distance error; (**b**) error percentage.

As shown in Figure 24, the largest vertical distance error of jogging in the plane is 0.61 m, the average error of absolute value is 0.01 m, and the variance of absolute value is 0.06 m. It can be seen from the statistical results of the two states that we can efficiently reduce the vertical distance error by leveraging gait information and creating a judgement model of the walking state.

#### 4.2.4. Experimental Estimation Step

For inertial sensors-based 3D localization, one of the important metrics is the accuracy of step length estimation. In this paper, we continuously collect data from four common walking states, normal walking, jogging, going upstairs, and going downstairs.

**Figure 25:**
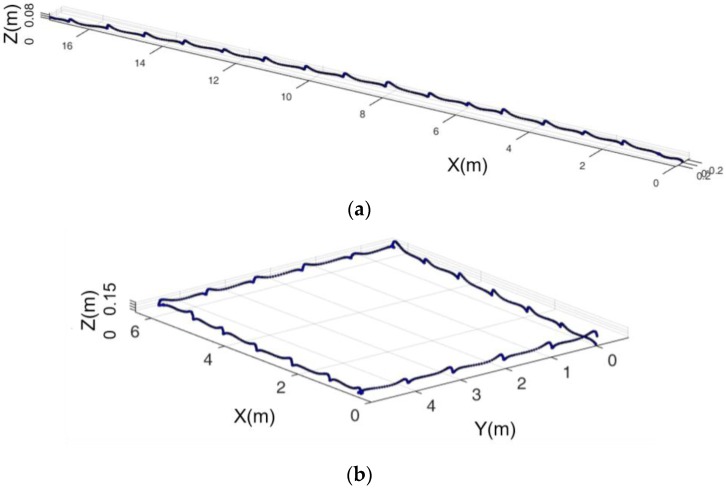
Track of walking on a horizontal plane: (**a**) walking straight; (**b**) walking along square.

Figure 25a shows an example of walking in a straight line. The real and estimated distance of the path is 19.2 m and 17.3 m, respectively, which means that our system can achieve an accuracy of 90.1%. Figure 25b shows an example of walking along a rectangle with a length of 6 m and a width of 7 m. It can be seen from the figure that these substantially coincide with the actual length and width. The following figure gives detailed statistics on the walking accuracy in the plane.

**Figure 26:**
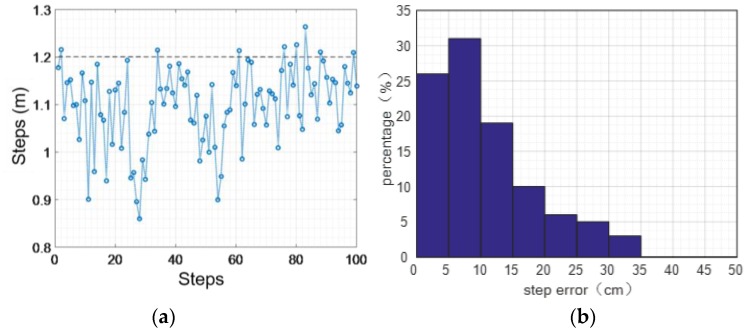
Normal walking step statistics: (**a**) distance error; (**b**) error percentage.

Figure 26 shows the statistics of step error while normal walking. In the condition of normal walking, the average length of each step is 1.20 m. We pick 100 steps randomly from our experimental data for statistical analysis. The result shows that the maximum error is 0.34 m, the average error of absolute value is 0.11 m, the mean square error of step length error is 0.08 m, and the accuracy of step length estimation is 90.83% while walking normally along a horizontal plane.

**Figure 27:**
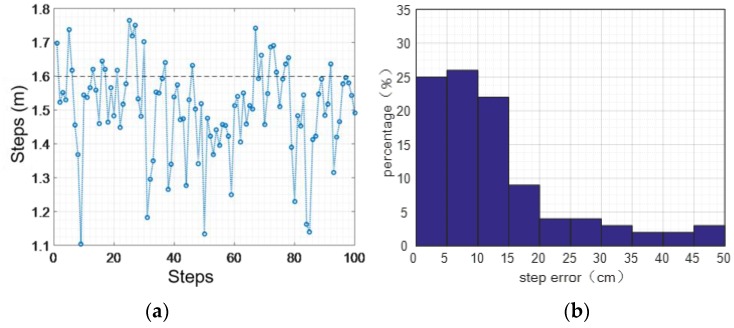
Jogging step statistics: (**a**) distance error; (**b**) error percentage.

Figure 27 shows the statistics of step errors while jogging. In the condition of jogging, the average length of each step is 1.60 m. We pick 100 steps randomly from our experimental data for statistical analysis. The result shows that the maximum error is 0.49 m, the average error of absolute value is 0.13 m, and the mean square error is 0.11 m; the accuracy of step length estimation is 91.87% while jogging along a horizontal plane.

**Figure 28:**
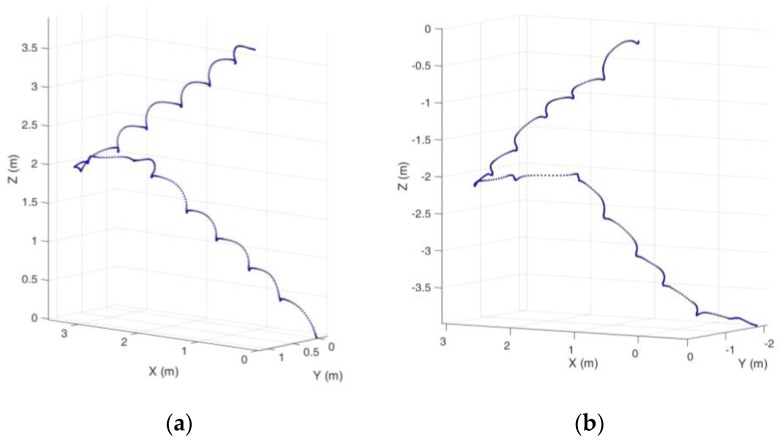
Upstairs and downstairs tracks: (**a**) upstairs; (**b**) downstairs.

Figure 28 shows an example of the trajectory of going upstairs or downstairs. The width of each step is 0.3 m and the height of each step is 0.16 m. When going up and down normally, we can regard alternating feet once as one step; the walking distance of each step is 0.60 m and the vertical height is 0.32 m. It can be seen from the trajectory in the figure that the statistics above substantially coincide with the trajectory of going upstairs or downstairs in reality.

**Figure 29:**
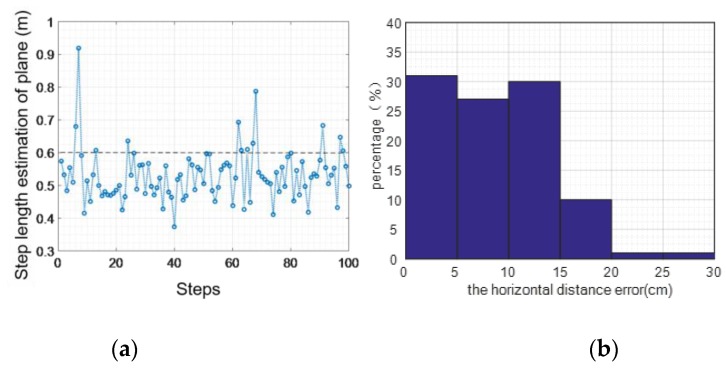
Horizontal step statistics (upstairs): (**a**) distance error; (**b**) error percentage.

Every step of walking upstairs can be regarded as both horizontal and vertical movement. Figure 29 shows an example of the statistical analysis of horizontal steps while going upstairs. It can be seen from the figure that the maximum error is 0.32 m, the average error of absolute value is 0.09 m, and the mean square error is 0.06 m. The accuracy of the horizontal step length when going upstairs is 90.83%.

**Figure 30:**
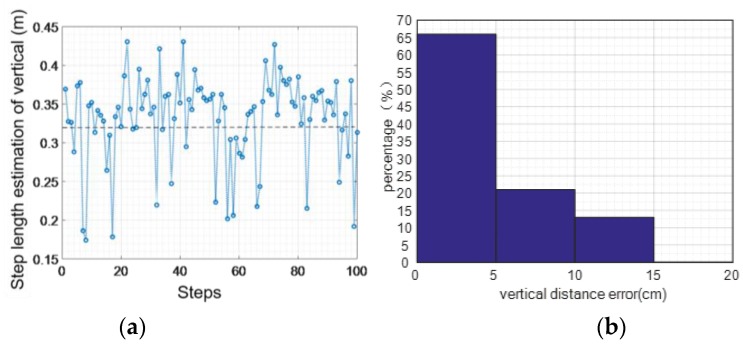
Vertical step statistics (upstairs): (**a**) distance error; (**b**) error percentage.

Figure 30 shows the statistical analysis of vertical movements per step while going upstairs. The maximum error is 0.14 m, the average error of absolute value is 0.04 m, and the mean square error is 0.03 m. The accuracy of vertical step length when going upstairs is 87.5%.

**Figure 31:**
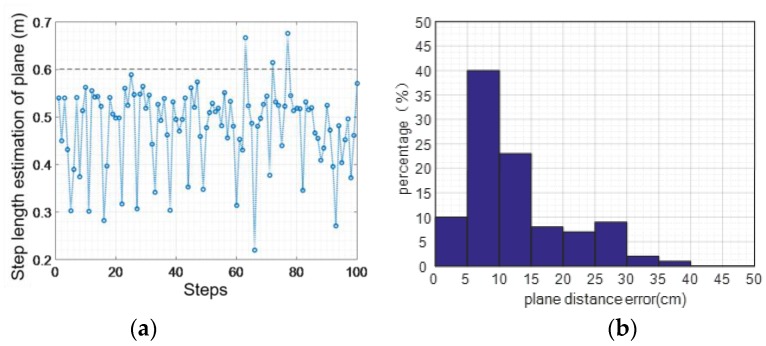
Horizontal step statistics (downstairs): (**a**) distance error; (**b**) error percentage.

Similarly, Figure 31 gives a statistical analysis of horizontal steps while going downstairs. We can find that the maximum error is 0.38 m, the average error of absolute value is 0.12 m, and the mean square error is 0.08 m; the accuracy of each horizontal step length is 80.0%.

**Figure 32:**
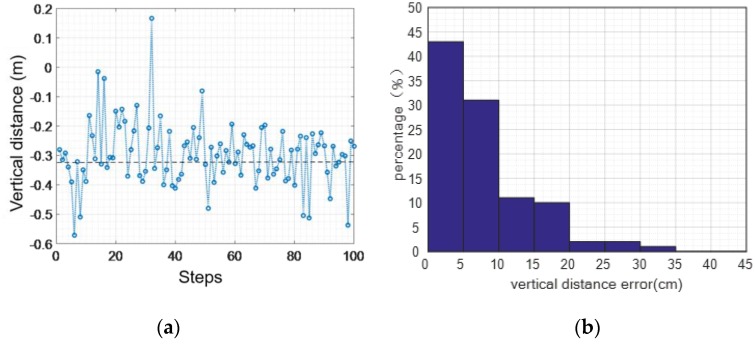
Vertical step statistics (downstairs): (**a**) distance error; (**b**) error percentage.

Figure 32 shows the statistical analysis of vertical steps while going downstairs. The maximum error is 0.30 m, the average error of absolute value is 0.07 m, and the mean square error is 0.06 m; the accuracy of each vertical step length is 79.1%.

Here we compare the performance of our tracking system with a method used in the literature [8]. First of all, we note that [8] was using high-precision fiber optic gyro sensors (DSP-1750) and the white noise is less than $18^{{}^{\circ}}h/\sqrt{Hz}$ at a normal temperature. In this paper we use low-cost inertial sensors (MPU-6050) and the white noise is $18^{{}^{\circ}}h/\sqrt{Hz}$ at the same temperature. The accuracy of the two sensors differ 22.5-fold. In order to ensure the parity of the experimental platform in the comparison, we perform a normalization process for the measurement error of both. The contrasting performance of the two systems is shown in Table 2.

The step estimation accuracy of our system is close to [8] most of the time, yet our system will achieve better results in the case of jogging.

#### 4.2.5. Heading Verification Experiment

**Figure 33:**
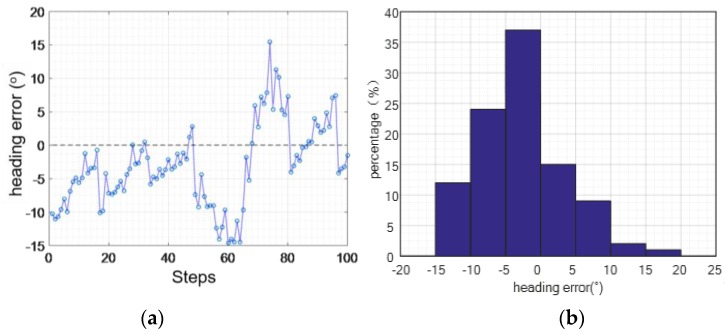
Walking heading angle error: (**a**) course error; (**b**) error percentage.

Course accuracy is one of the significant indicators of inertial sensors-based indoor 3D positioning. We evaluated this indicator by asking three participants to walk and jog straight along a 19.2-m path (forth and back 10 times). The statistical results are shown in Figure 33. We find that the mean value of the course error for every step is close to 0° in normal conditions, the maximum error of the yaw angle is 15.46°, the mean value of absolute error of the course angle is 5.65°, and the mean square error is 3.88°.

**Figure 34:**
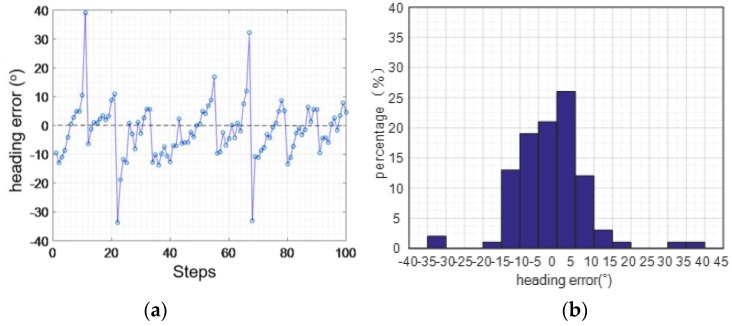
Jogging heading angle error: (**a**) course error; (**b**) error percentage.

Figure 34 shows the jogging heading angle error. We find that the maximum error is 39.13°, the average of the absolute value heading angle error is 7.09°, and the mean square error of the heading angle is 6.94°.

#### 4.2.6. Overall Effect of Indoor 3D Positioning

**Figure 35:**
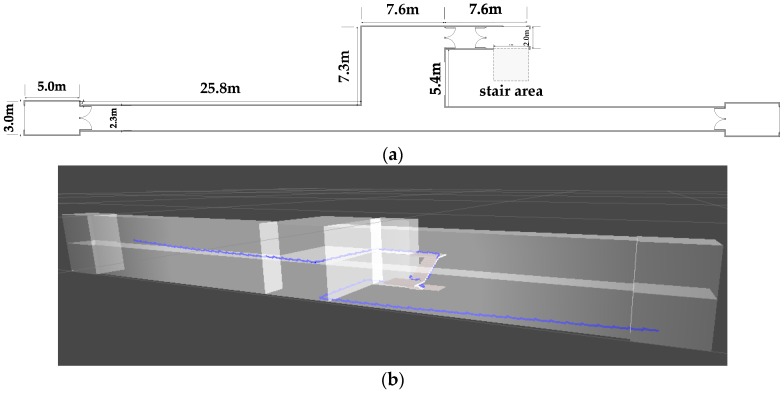
3D positioning: (**a**) corridor structure; (**b**) 3D positioning trajectory in 3D modeling.

This section shows the overall effect of 3D positioning. Figure 35a shows the structure of every floor of the building. As shown in Figure 35b, the trajectory of 3D positioning for 5 min is basically in conformity with the actual trajectory, which means that our system can provide accurate 3D positioning.

## 5. Conclusions

In this paper, we propose a shoe sensing-based 3D tracking solution aiming to achieve real-time indoor 3D positioning leveraging low-cost inertial sensors that can be easily attached to the user’s shoes. The proposed system reduces the cumulative errors caused by the sensors’ internal bias using the linear property of the cumulative acceleration error drift. In addition, we also propose a walking state classification model that is able to distinguish different moving status including normal walking/jogging, going upstairs, and going downstairs. A real-time 3D trajectory dynamic map is also built relying on unity3D and the system’s tracking results. Extensive experiments have been conducted using a low-cost sensor module MPU6050, which demonstrates that the proposed system could accurately track users’ 3D indoor positions and moving trajectories.

Large-scale deployment of the shoe sensing-based real-time localization requires careful consideration of a set of key networking metrics (e.g., throughput, delay, and energy efficiency). Several studies (e.g., [34,35]) redesigned the networking scheme in terms of these critical metrics for large-scale WSN and IOT networks. We leave the study of large-scale shoe sensing deployment to our future work.

## Figures and Tables

**Table 1:** The average and standard deviation of $\theta^{\prime }$.

Types	Average Value (°)	Standard Deviation (°)
plane	3.935	6.435
upstairs	107.904	36.465
downstairs	−89.464	34.907

**Table 2:** Step estimation normalized error comparison.

	Error (DSP-1750 [8])	Error (MPU-6050)
walking	0.19%	0.40%
jogging	6.25%	0.36%
upstairs	0.30%	0.56%
downstairs	0.90%	0.88%
